# Antibiotic choice for Group B Streptococcus prophylaxis in mothers with reported penicillin allergy and associated newborn outcomes

**DOI:** 10.1186/s12884-023-05697-0

**Published:** 2023-05-30

**Authors:** Josephine B. Snider, Leena B. Mithal, Jason H. Kwah, Nathaniel J. Rhodes, Moeun Son

**Affiliations:** 1 Department of Pediatrics, Division of Hospital-Based Medicine, Ann and Robert H. Lurie Children’s Hospital of Chicago and Feinberg School of Medicine, Northwestern University, Chicago, IL USA; 2 Department of Pediatrics, Division of Infectious Diseases, Ann and Robert H. Lurie Children’s Hospital of Chicago and Feinberg School of Medicine, Northwestern University, Chicago, IL USA; 3 Department of Medicine, Section of Rheumatology, Allergy, and Immunology, School of Medicine, Yale University, New Haven, CT USA; 4 Midwestern University Chicago College of Pharmacy,Center of Pharmacometric Excellence, Midwestern University, Downers Grove, IL USA; 5 Department of Obstetrics, Gynecology, and Reproductive Sciences, Section of Maternal-Fetal Medicine, School of Medicine, Yale University, 333 Cedar Street, P.O. Box 208063, New Haven, CT 06520 USA

**Keywords:** Group B streptococcus, GBS prophylaxis, Neonatal sepsis, Penicillin allergy

## Abstract

**Objective:**

To evaluate the choice of antibiotic used for intrapartum Group B Streptococcus (GBS) prophylaxis in pregnant individuals with reported penicillin allergies compared to those without reported penicillin allergies and investigate whether there are associated differences in neonatal outcomes.

**Study Design:**

This retrospective cohort study included mother-infant dyads of GBS positive pregnant individuals who labored and delivered newborns ≥ 35 weeks of gestation at a high-volume urban hospital (2005–2018). The type of antibiotic administered to the mothers for GBS prophylaxis (beta-lactam prophylaxis defined as penicillin-class drug or cefazolin; alternative prophylaxis defined as vancomycin or clindamycin) was compared between those with a penicillin allergy documented in their medical record versus those who did not. Neonatal outcomes included number of postnatal blood draws, antibiotic administration, neonatal intensive care unit (NICU) admission, bacteremia, and hospital length of stay and were compared between groups. Bivariable and multivariable analyses were performed.

**Results:**

Of 11,334 mother-infant pairs, 1170 (10.3%) mothers had a penicillin allergy documented in their medical record. Of them, 49 (4.2%) received a penicillin, 259 (22.1%) received cefazolin, 449 (38.4%) received clindamycin, and 413 (35.3%) received vancomycin. Patients with a reported penicillin allergy were significantly more likely to receive alternative GBS prophylaxis compared to those without penicillin allergy (73.7% vs. 0.2%, p < 0.01). Neonates of patients who received alternative GBS prophylaxis were significantly more likely to undergo a postnatal lab draw compared to neonates of patients who received beta-lactam antibiotics (20.8% vs. 17.3%, OR 1.25 (95% CI 1.08–1.46)). This significant association persisted after adjusting for potential confounders (aOR 1.23, 95% CI 1.06–1.43). There were no other significant differences seen in other newborn outcomes.

**Conclusion:**

Pregnant individuals who report a penicillin allergy were more likely to receive alternative antibiotics for GBS prophylaxis compared to those without a penicillin allergy. This was associated with an increased frequency of postnatal blood draws among neonates of mothers with a reported penicillin allergy.

**Brief summary:**

Administration of alternative intrapartum antibiotic prophylaxis with vancomycin or clindamycin is common in individuals with self-reported penicillin allergy, and maternal alternative antibiotic administration may impact neonatal care, particularly via increased lab draws.

## Introduction

Vertical transmission of Group B Streptococcus (GBS) infection is the most common cause of early onset neonatal sepsis and can lead to significant morbidity and mortality in the newborn [1, 2]. To date, the only effective strategy to reduce the risk of early onset neonatal GBS disease is intrapartum antimicrobial prophylaxis in mothers who are known or suspected to be colonized with GBS [3–5]. Implementation of national guidelines [2] has led to a drastic decline of early onset neonatal GBS sepsis, with a nearly 8-fold decrease since 1990 [6–8]. Beta-lactam antibiotics, specifically penicillin and cephalosporins, are considered first-line for GBS prophylaxis because they are highly effective, have a narrow spectrum of activity, and are less likely to result in antibiotic-related complications [2]. Unfortunately, a penicillin allergy is commonly reported with roughly 10% of the U.S. population reporting a history of a penicillin allergy, although most are unverified [9]. The existing literature shows that there is significant variability in appropriate antibiotic selection for intrapartum GBS prophylaxis, particularly for pregnant individuals who report a penicillin allergy [10, 11]. Alternative antibiotics, such as clindamycin and vancomycin, are often used for patients who report a penicillin allergy even though most have a low-risk allergy or do not have a confirmed true allergy [12]. Inappropriate use of beta-lactam alternatives has been associated with worse outcomes for non-pregnant patients and increased healthcare utilization costs [13–15]. In pregnant patients, being labeled with a penicillin allergy has been associated with increased maternal morbidities and longer length of hospital stay [16]. However, less is known about the impact of a maternal beta-lactam allergy label on the neonate in the setting of GBS prophylaxis.

While studies have shown that alternative antibiotics such as clindamycin and vancomycin can reach therapeutic levels in the fetus, they have not been demonstrated to effectively prevent neonatal GBS disease [17, 18] and may in fact be associated with increased rates of neonatal early-onset GBS disease [6]. Therefore, the American Academy of Pediatrics (AAP) does not consider these antibiotics to be adequate prophylaxis [4]. If a pregnant mother receives alternative antibiotics and then experiences any risk factors for neonatal GBS infection, such as fever, preterm labor or prolonged rupture of membranes, the neonate must be considered at higher risk of early onset GBS infection [4]. During the time of our data collection (2005–2018), the Centers for Disease Control and Prevention (CDC) recommended that this subset of higher risk infants should undergo laboratory testing to assess for infection, with some of these infants going on to receive empiric antibiotics if labs were abnormal [2].

Therefore, the objective of this study was to evaluate the type of intrapartum antibiotics used for GBS prophylaxis in pregnant individuals with and without a documented penicillin allergy and to investigate any associated differences in the medical management of their newborn infants.

## Materials and methods

This is a retrospective cohort study of mothers with antenatally diagnosed GBS colonization who received intrapartum antibiotic prophylaxis and delivered a live born neonate at ≥ 35 weeks of gestation at Northwestern Memorial Hospital in Chicago, Illinois, from January 1, 2005 to March 2, 2018. All potentially eligible individuals were identified through a query of hospital electronic medical records (EMR). This study period was chosen as there were no significant changes to the EMR system during this time interval. We systematically identified patients with known GBS colonization during pregnancy by searching templated labor and delivery admission notes that routinely document the result of a known GBS culture (rectovaginal or urine) and laboratory results, and only included individuals who had a known positive GBS result. Antibiotic susceptibility results from positive GBS culture were not available. Patients were included if they were at least 18 years of age, received at least one intrapartum dose of an antibiotic for the indication of GBS prophylaxis, and if they delivered a live born singleton infant at ≥ 35 weeks of gestation. This gestational age threshold was chosen because institutional protocol requires neonatal intensive care unit (NICU) admission for all neonates born at < 35 weeks of gestation. Patients were excluded if they were suspected to have intrapartum chorioamnionitis because it was institutional practice during the study period for their neonates to routinely have blood draws to assess complete blood count (CBC) and blood cultures, and to receive at least 24 h of IV antibiotics or until the blood tests resulted. Chorioamnionitis was defined by the presence of billing diagnosis codes ICD-9 658.4 (Infection of amniotic cavity) or ICD-10 O41.1 (Infection of amniotic sac and membranes), or the presence of the diagnosis of “chorioamnionitis/sepsis” in the templated delivery note, or the presence of an intrapartum temperature ≥ 100.4 F and the administration of therapeutic doses of antibiotic agents typically used to treat chorioamnionitis (i.e., ampicillin and gentamicin). Lastly, mother-neonate dyads for whom exposure or outcome data were missing were excluded.

Demographic, clinical, laboratory, and pharmacy data was collected through EMR query through the Northwestern University Electronic Data Warehouse, and a combination of manual chart review and pharmacy antibiotic medication administration records. The exposure of interest was maternal penicillin allergy documented at the time of admission during their delivery hospitalization encounter. It is standard institutional procedure that a patient’s allergy history is obtained, reviewed, and documented in the electronic medical chart at the time of their delivery hospitalization admission. Individuals were considered to have a documented penicillin allergy if they reported an allergy to any penicillin-class drug including penicillin, ampicillin, ampicillin-sulbactam, amoxicillin, dicloxacillin, methicillin, nafcillin, oxacillin, or piperacillin-tazobactam. Details and severity of historic allergic reaction were not reliably documented in the medical record. It is notable that during the study period, it was rare for patients to undergo formal penicillin allergy verification testing during pregnancy at our institution, and therefore it is unlikely that the documented penicillin allergy was confirmed to be a true allergy in most cases.

Demographic and clinical data collected included maternal age, race and ethnicity, parity, and gestational age at delivery. The presence of a documented maternal cephalosporin allergy was also collected. Patients were considered to have a documented cephalosporin allergy if there was a documented allergy to any generation cephalosporin available in the United States. Obstetric data abstracted included length of time of ruptured membranes (irrespective of spontaneous or artificial rupture) prior to delivery. The receipt of any prophylactic doses of antibiotics for the indication of GBS prophylaxis prior to delivery was determined. If a woman received more than one antibiotic class type for the indication of GBS prophylaxis during her labor (e.g., cefazolin and vancomycin), the receipt of multiple antibiotic class types was noted but the broader-spectrum antibiotic (e.g., vancomycin over cefazolin) was used to assess the primary outcome. Beta-lactam antibiotics including penicillin, ampicillin, and cefazolin were considered first-line antibiotics. Clindamycin and vancomycin were considered alternative antibiotics. Baseline neonatal data abstracted included sex and birth weight. Small-for-gestational age (SGA) birthweight was defined as birthweight < 10th percentile according to the infant sex-specific methodology published by Aris et al. [19].

Patients with a documented penicillin allergy were compared to those without a documented penicillin allergy. Among the mothers, we examined and compared receipt of alternative antibiotics (clindamycin or vancomycin) for GBS prophylaxis between groups. Among their neonates, the outcomes compared were the number of blood draws for laboratory tests (specifically CBC, C-reactive protein (CRP), and blood culture), the frequencies of positive blood cultures, administration of antibiotics, admissions to the NICU, and neonatal length of stay. Neonatal outcomes were assessed until birth hospitalization discharge.

Categorical variables were analyzed with Chi-square tests or Fisher’s exact tests, and continuous variables were analyzed using Mann Whitney U tests given non-normal distributions. All hypothesis testing was two-tailed and p < 0.05 was used to define statistical significance. Logistic regression was also performed to present crude odds ratios and 95% confidence intervals for the categorical neonatal outcomes. Multivariable analyses were performed adjusting for potential confounders using pre-delivery baseline characteristics with p < 0.05 on bivariable analyses. Statistical analyses were performed using Stata version 15.1 (StataCorp, College Station, TX). Approval for this study was obtained from the Northwestern University Institutional Review Board with a waiver of informed consent prior to its initiation. All study procedures were performed in accordance with relevant guidelines and reported following STROBE guidelines.

## Results

**Fig. 1:**
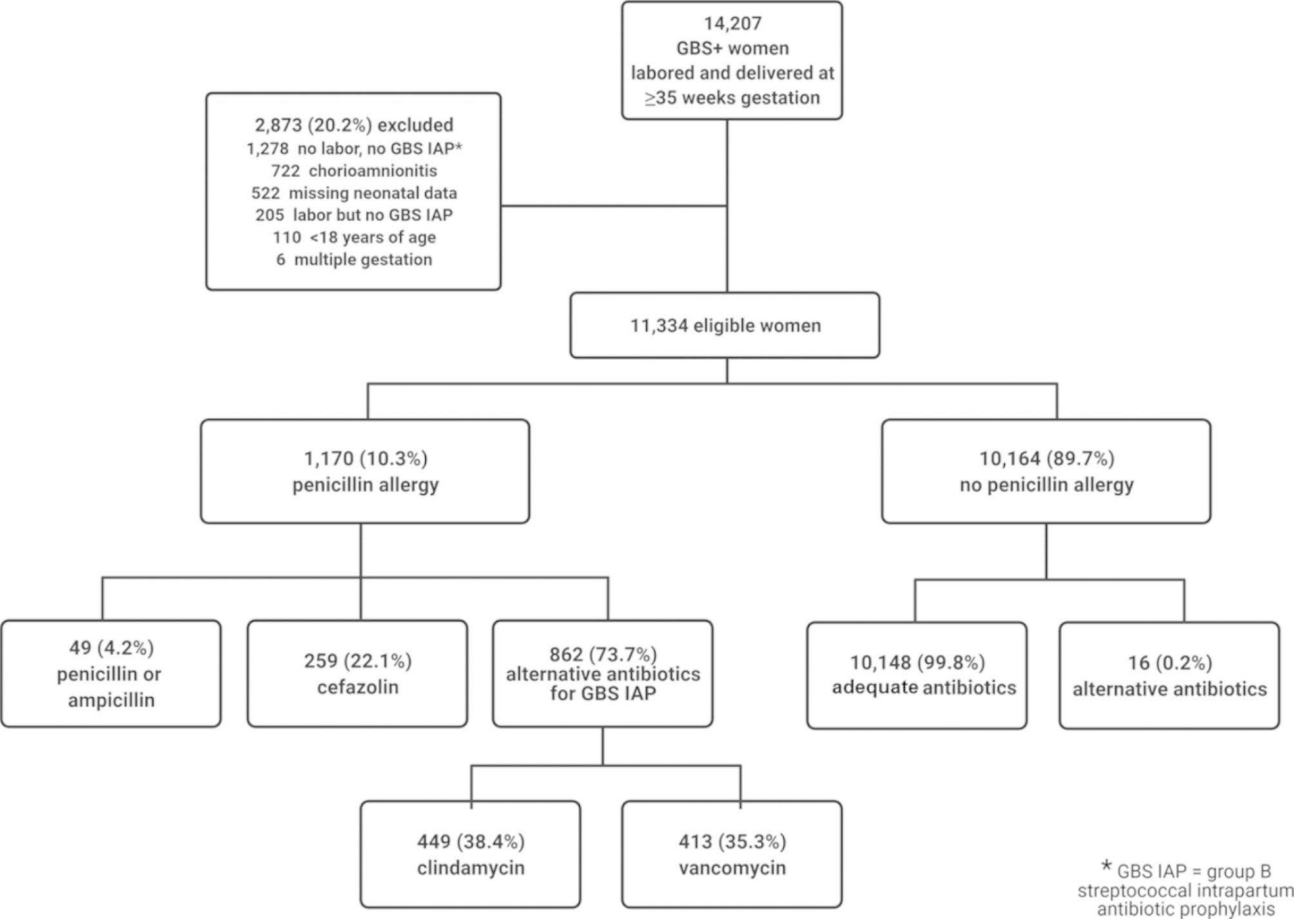
Legend: Study cohort flow diagram

Of the 11,334 mother-infant dyads who met study eligibility criteria, 1170 (10.3%) had a documented penicillin allergy during their delivery hospitalization encounter (Fig. 1). The rate of a documented penicillin allergy was stable over time (annual rate range 9.4–14.2%). Cephalosporin allergy was documented in 51 (4.4%) patients with documented penicillin allergy compared to 93 (0.9%) patients without documented penicillin allergy. Baseline characteristics of analyzed mother-infant pairs are shown in Table 1. Mothers with a documented penicillin allergy were significantly more likely to be older, be of non-Hispanic White race, and have a documented cephalosporin allergy compared to mothers who did not have a documented penicillin allergy.

**Table 1:** Demographic and baseline characteristics of mothers with known positive Group B Streptococcus carrier status and their neonates

Characteristic	Documented penicillin allergy (n = 1170)	Absence of documented penicillin allergy (n = 10164)	p-value
Maternal age, years			< 0.01
<20	19 (1.6)	216 (2.1)	
20–34	768 (65.6)	7169 (70.5)	
>=35	383 (32.7)	2779 (27.3)	
Race/Ethnicity			< 0.01
Non-Hispanic White	729 (62.3)	5237 (51.5)	
Non-Hispanic Black	119 (10.2)	1218 (12.0)	
Hispanic	108 (9.2)	1812 (17.8)	
Asian	77 (6.6)	731 (7.2)	
Unknown or mixed race	137 (11.7)	1166 (11.5)	
Multiparous	415 (35.5)	3883 (38.2)	0.07
Gestational age at delivery (weeks)	39.4 (38.6–40.2)	39.4 (38.6–40.3)	0.12
Preterm birth 35^0/7^- 36^6/7^ weeks of gestation	32 (2.7)	247 (2.4)	0.52
Duration of rupture of membranes (hours)^a^	6 (3–11)	6 (2–11)	0.07
Intrapartum cesarean	204 (17.4)	1342 (13.2)	< 0.01
Delivery hospitalization postpartum length of stay (hours)	55.8 (50.2–64.9)	55.2 (49.8–63.1)	< 0.01
Female neonate	588 (50.3)	5023 (49.4)	0.59
SGA birthweight^b^	117 (10.0)	978 (9.6)	0.66

Data are presented as n (%) or median (interquartile range) unless otherwise specified

SGA = small for gestational age

^a^Data available for 11,129 mothers (1157 with a documented penicillin allergy and 9972 without a documented penicillin allergy)

^b^Data available for 11,324 neonates (1169 whose mothers had a documented penicillin allergy and 10,155 whose mothers did not have a documented penicillin allergy). SGA status based on birthweight < 10th percentile using neonatal sex-specific methodology by Aris et al. [12]

Mothers with a documented penicillin allergy were significantly more likely to receive alternative antibiotics for intrapartum GBS prophylaxis (p < 0.001). Almost all patients (99.8%) without a documented penicillin allergy received penicillin or ampicillin for GBS prophylaxis during labor. All 16 patients without a documented penicillin allergy who received alternative antibiotics had a documented cephalosporin allergy. Among patients with a documented penicillin allergy during their delivery hospitalization encounter, only 259 (22.1%) received intrapartum cefazolin for GBS prophylaxis, while 449 (38.4%) patients with a documented penicillin allergy received clindamycin and 413 (35.3%) received vancomycin. There were 49 (4.2%) patients who received penicillin or ampicillin for GBS prophylaxis despite having a penicillin allergy documented in their medical chart.

The neonates of individuals with a documented penicillin allergy were significantly more likely to have their blood drawn postnatally for laboratory tests compared to those of without a documented penicillin allergy (20.8% vs. 17.3%; OR 1.25 (95% CI 1.08–1.46)). Neonates of individuals with reported penicillin allergy were more likely to have a CBC (20.6% vs. 17.2%) and blood culture (17.9% vs. 14.2%) drawn compared to neonates of mothers without a reported penicillin allergy. However, the frequency of positive blood culture results was similar between the two groups (Table 2). There was one positive blood culture for GBS in each group for a total of 2 out of 19 positive blood cultures. The median birth hospitalization length of stay was marginally longer (56.6 h vs. 55.6 h; p < 0.01) for infants of mothers with a reported penicillin allergy compared to infants of mothers without reported penicillin allergy (Table 2). This finding was specific to infants born via intrapartum cesarean (n = 1546) in which infants of mothers with a reported penicillin allergy had a significantly longer median birth hospitalization length of stay compared to those born to mothers without a reported allergy (91.5 (interquartile range (IQR) 79.6-104.2) vs. 88.9 (IQR 76.8-100.3) hours, p = 0.02). Among infants born via vaginal delivery, the median birth hospitalization length of stay was similar (54.3 (IQR 49.2–61.0) vs. 54.1 (IQR 49.2–60.5) hours, p = 0.61). Other neonatal outcomes including NICU admission and rates of positive blood culture were not statistically different between the groups, though infants of mothers with penicillin allergy were numerically more likely to receive postnatal IV antibiotics compared to mothers without documented allergies (6.8% vs. 5.5%) (Table 2).

**Table 2:** Neonatal outcomes among offspring of mothers with a documented penicillin allergy versus those without a documented penicillin allergy

Outcome	Documented maternal penicillin allergy (n = 1170)	Absence of documented maternal penicillin allergy (n = 10164)	p-value	OR (95% CI)	aOR (95% CI)^c^
Blood drawn for lab test^a^	243 (20.8)	1758 (17.3)	< 0.01	1.25 (1.08–1.46)	1.23 (1.06–1.43)
Complete blood count	241 (20.6) 40 (3.4)	1751 (17.2) 331 (3.3)			
C-reactive protein	209 (17.9)	1447 (14.2)			
Blood culture					
Positive blood culture^b^	3 (3.3)	16 (2.5)	0.64	1.71 (0.56–5.21)	NA^d^
Receipt of postnatal antibiotics	80 (6.8)	563 (5.5)	0.07	1.25 (0.98–1.59)	1.20 (0.94–1.53)
NICU admission	101 (8.6)	775 (7.6)	0.22	1.14 (0.92–1.42)	1.11 (0.89–1.38)
Birth hospitalization length of stay, hours	56.6 (50.6–66.6)	55.6 (50.0-64.4)	< 0.01	NA	NA

Data presented as n (%), median (interquartile range), p-values, and odds ratio (95% confidence intervals)

^a^ A single blood draw may have encompassed a single or multiple laboratory tests

^b^ Frequencies calculated as the number of positive blood cultures divided by total number of blood cultures performed

^c^ Adjusted for maternal age at delivery, maternal race and ethnicity, and intrapartum cesarean delivery

^d^ Modeling not performed due to low frequencies of events

In multivariable analyses, after adjusting for maternal age at delivery, maternal race and ethnicity, and intrapartum cesarean delivery, the significant associated risk of postnatal blood draw for the newborns persisted with aOR 1.23 (1.06–1.43). The other non-significant differences in newborn outcomes remained non-significant in multivariable analyses (Table 2).

## Discussion

Despite the CDC’s recommendation to treat penicillin allergic pregnant individuals with cefazolin for GBS prophylaxis, the majority (73.7%) of patients with a documented penicillin allergy in our cohort were treated with clindamycin or vancomycin, both considered alternative treatment for GBS prophylaxis. This is consistent with prior smaller studies that have showed obstetric care providers have an insufficient understanding of appropriate antibiotic selection for GBS prophylaxis in penicillin allergic patients [10, 11, 20]. Our findings in this large study demonstrates a notable deviation from a well-established best practice guideline and has several concerning implications, particularly relating to effects of poor antibiotic stewardship and rising antimicrobial resistance rates. GBS resistance to clindamycin is already a significant problem, with the CDC reporting 47.3% of isolates to be resistant [21]. The most recent update in 2019 to the ACOG guideline addresses this by now only recommending the use of clindamycin for GBS prophylaxis when a culture is obtained and shows a susceptible strain. This illustrates how effective antibiotic choices and reliability of empiric therapy are decreasing over time. The improper use of clindamycin and vancomycin also leads to broader antimicrobial coverage than indicated (e.g., anaerobic coverage with clindamycin) and may have effects on the maternal and perhaps perinatally-acquired neonatal microbiome [22, 23]. Broader coverage may also lead to increased antibiotic resistance among other common colonizing bacteria, such as Staphylococcus aureus (rising clindamycin resistance rates) and enterococcus species (e.g., vancomycin resistant enterococcus) [24, 25]. Furthermore, compared to beta-lactam antibiotics, alternative antibiotics are more costly and more toxic, with increased risks for maternal nephrotoxicity and Clostridium difficile infections [26–28].

Our study also showed that the use of clindamycin and vancomycin for GBS prophylaxis had consequences for the newborn during the study period. Neonates of mothers who received clindamycin or vancomycin had an increased rate of blood draws (specifically, CBC and blood culture) compared to neonates of mothers who received penicillin or cefazolin. These lab draws would likely not have been medically indicated if their mothers had received first-line GBS prophylaxis during labor. Venous blood draws are distressing both to the infant and parents and should be avoided whenever possible. Although as of 2019, enhanced clinical is recommended for infants born > 35 weeks gestational age to mothers who received inadequate antibiotic prophylaxis rather than routine laboratory evaluation, the study illustrates deviation from “routine neonatal care” if alternative antibiotics without equal efficacy data for neonatal sepsis prevention are utilized.

It should also be considered that broader-spectrum antibiotics like clindamycin and vancomycin may have direct negative impacts on the health of a neonate. Studies have shown that transplacental passage of intrapartum vancomycin and clindamycin can reach therapeutic levels in the fetus [17, 18, 29]. This raises the question of what impact this could have on the fetus, such as damage to the already fragile kidneys of a neonate or alteration of the perinatal and developing neonatal microbiome after birth. For example, it has been shown that infants born to mothers who received intrapartum antibiotics had altered gut microbiomes at 3 months of age compared to infants who were not exposed to intrapartum antibiotics [30]. Further investigations to explore the potential adverse effects of vancomycin and clindamycin exposure for the infant, particularly the risk of nephrotoxicity and alteration of the intestinal microbiome, is warranted given the high risk of exposure to these antibiotics.

A significant strength of this study is the large sample size and findings can likely be extrapolated to other large academic center birthing hospitals, although it is less clear how our findings translate to practice in smaller community hospitals. This study was limited by being a retrospective observational study. In addition, we were unable to extrapolate the severity of penicillin allergy, as most patients did not have any specific reaction listed in their medical record. Therefore, we do not know what percentage of the cohort had a high risk for anaphylaxis allergy history and appropriately received alternative antibiotics. However, as previously mentioned, most reported reactions to penicillin (even those with penicillin allergy of mild-moderate severity) are at very low risk for anaphylaxis. We were also unable to extract data on whether patients had undergone penicillin allergy verification testing (i.e., penicillin skin test or oral challenge test), but such cases were likely to be very rare as this was not routine practice during the study period. Lastly, with regard to neonatal outcomes, there were other characteristics that were not accounted for such as maternal diabetes and Apgar scores, which may have affected the likelihood of blood draws, NICU admission or length of stay.

Our study showed that most individuals in our cohort with a documented penicillin allergy received alternative broad-spectrum antibiotics, and their neonates were more likely to have laboratory blood draws (deviation from routine clinical care) compared to those of mothers without a documented penicillin allergy. Most individuals with documented penicillin allergy should be able to safely receive penicillin or cefazolin, unless specifically deemed to be at high risk for anaphylaxis. Determining why medical providers disproportionately choose vancomycin or clindamycin over cefazolin will be important going forward to improve adherence to current best-practice GBS prophylaxis guidelines. There is a clear need for implementing healthcare provider education regarding antibiotic selection in penicillin allergic patients and the role of penicillin testing in pregnancy to improve appropriate and targeted antibiotic choice and impact on newborn infants born to GBS positive mothers [31, 32]. Over 90% of patients who report a history of penicillin allergy are not truly allergic [33, 34]. As such, in August 2019 (notably after our study data was collected), the ACOG Committee Opinion on the prevention of early-onset GBS disease in newborns was updated and now “encourages the expansion of the use [of penicillin allergy skin testing] in obstetric patients”, as verification of a reported penicillin allergy among pregnant individuals [35]. Penicillin testing during pregnancy remains underutilized, yet our data suggests that there is deviation from routine clinical care and potential adverse impact to neonates born to individuals with penicillin allergy who are treated with alternate GBS intrapartum prophylaxis antibiotics. Provider education and incorporation of screening and testing for penicillin allergy should be considered as part of routine prenatal care to optimize care of the mother-infant dyad.

## Data Availability

The datasets used and/or analyzed during the current study are available from the corresponding author on reasonable request.
